# JC and BK virus sequences are not detectable in leukaemic samples from children with common acute lymphoblastic leukaemia

**DOI:** 10.1038/sj.bjc.6690783

**Published:** 1999-11

**Authors:** J MacKenzie, J Perry, A M Ford, R F Jarrett, M Greaves

**Affiliations:** LRF Virus Centre, Department of Veterinary Pathology, University of Glasgow, Glasgow G61 1QH, UK; LRF Centre, Institute of Cancer Research, Chester Beatty Laboratories, London SW3 6JB, UK

**Keywords:** leukaemia, JC virus, BK virus, aetiology

## Abstract

Epidemiological evidence suggests that childhood leukaemia, and possibly common acute lymphoblastic leukaemia in particular, may have an infectious aetiology. Smith (1997 *J Immunother***20**: 89–100) recently suggested that the critical infectious event occurs during pregnancy, and identified the polyoma virus JC as a candidate agent. In the present study we investigated whether genomes from the JC virus, and closely related BK virus, could be detected in leukaemic cells. No positive results were obtained suggesting that JC virus is unlikely to play a direct role in leukaemogenesis. © 1999 Cancer Research Campaign


*British Journal of Cancer* (1999) **81**(5), 898–899
© 1999 Cancer Research Campaign
Article no. bjoc.1999.0783

Short Communication

### JC and BK virus sequences are not detectable in leukaemic samples from children with common acute lymphoblastic leukaemia

**J MacKenzie[1], J Perry[1], AM Ford[2], RF Jarrett[1] and M Greaves[2]**

[1]LRF Virus Centre, Department of Veterinary Pathology, University of Glasgow, Glasgow G61 1QH, UK; [2]LRF Centre, Institute of Cancer Research, Chester Beatty Laboratories, London SW3 6JB, UK

**Summary** Epidemiological evidence suggests that childhood leukaemia, and possibly common acute lymphoblastic leukaemia in particular, may have an infectious aetiology. Smith (1997 *J Immunother* **20**: 89–100) recently suggested that the critical infectious event occurs during pregnancy, and identified the polyoma virus JC as a candidate agent. In the present study we investigated whether genomes from the JC virus, and closely related BK virus, could be detected in leukaemic cells. No positive results were obtained suggesting that JC virus is unlikely to play a direct role in leukaemogenesis. © 1999 Cancer Research Campaign

**Keywords:** leukaemia; JC virus; BK virus; aetiology

There is a body of epidemiological evidence which suggests that childhood leukaemia, and common acute lymphoblastic leukaemia (cALL), in particular, may have an infectious aetiology. Several models for disease development involving infectious agents have been proposed and are reviewed in detail elsewhere (Kinlen et al, 1990; Greaves and Alexander, 1993; Kinlen, 1995; Greaves 1997). In 1997, Smith put forward the idea that the critical infectious event occurs in utero and suggested that the polyoma virus JC was a candidate agent (Smith, 1997). Most individuals are infected with JC virus in childhood and primary infection is usually asymptomatic but the virus can cause progressive multifocal encephalopathy in immunosuppressed individuals (Perrons et al, 1996). Reactivation of latent viral infection has been described in pregnancy and delayed infection in developed, as compared to developing, countries may result in primary infection during reproductive years (reviewed in Smith, 1997). The virus has the ability to infect B-cells, encodes a T antigen similar to that of other polyoma viruses and has been shown to have oncogenic potential in animal model systems (Smith, 1997). In order to test the hypothesis that JC virus is directly associated with the aetiology of cALL, we investigated whether genomes from JC virus, and the related polyoma virus BK, are detectable in leukaemic blasts from children with cALL.

### MATERIALS AND METHODS

Pre-treatment samples from 15 cases, nine males and six females, age range 1–12 years were investigated. Leukaemic cells in all cases fulfilled the phenotypic criteria for diagnosis of cALL ($CD10^+$, $CD19^+$, $TdT^+$, FAB L1 or L2) and > 90% of the mononuclear cells were blast cells. DNA was extracted from peripheral blood or bone marrow mononuclear cell fractions and subjected to polymerase chain reaction (PCR).

*Received 14 January 1999*
*Revised 17 March 1999*
*Accepted 29 March 1999*

*Correspondence to:* RF Jarrett

PCR primer sequences were derived from the gene encoding the large T antigen of JC and BK viruses and the human β-globin gene (Saiki et al, 1988; Perrons et al, 1996). A common 5′ primer was used in conjunction with a 3′ primer specific for each of the two viruses. These primers were originally described by Perrons et al (1996), as the inner primer set in a nested PCR reaction; however, titration experiments (see below) indicated that the single-round PCR used was sufficiently sensitive for the purposes of this study. β-Globin PCR was used to confirm that samples contained amplifiable DNA.

PCR reactions contained 1 μg of template DNA, PCR buffer containing 1.5 mM magnesium chloride, 200 μM dNTPs, 1 μM primers and 5 units of Amplitaq thermostable polymerase (Perkin-Elmer Biosystems, Warrington, UK). Hot-start PCR was accomplished by the addition of TaqStart antibody (Clontech UK Ltd, Hampshire, UK). Thermal cycling was performed on a Perkin-Elmer Thermal Cycler (Perkin-Elmer Biosystems) using the following conditions: initial denaturation at 95°C for 5 min, followed by 40 cycles of ramping to 94°C over 1 min; 94°C for 30 s; cooling to 55°C over 2 min; 55°C for 10 s; heating to 72°C over 1 min; 72°C for 30 s, followed by a final extension step at 72°C for 7 min. PCR products were analysed by electrophoresis on 8% polyacrylamide gels followed by electroblotting and hybridization with $^{32}$P-labelled oligonucleotide probes. Positive controls consisted of a urine sample known to contain JC virus and conditioned medium from a culture of BK virus.

In order to test the sensitivity of the PCR reactions, DNA fragments obtained from the positive control reactions were cloned into plasmid. Serial tenfold dilutions of the cloned fragments in placental DNA were performed and subjected to PCR. Both assays had the ability to detect ten copies of the relevant viral sequence in a background of 1-μg high molecular weight DNA.

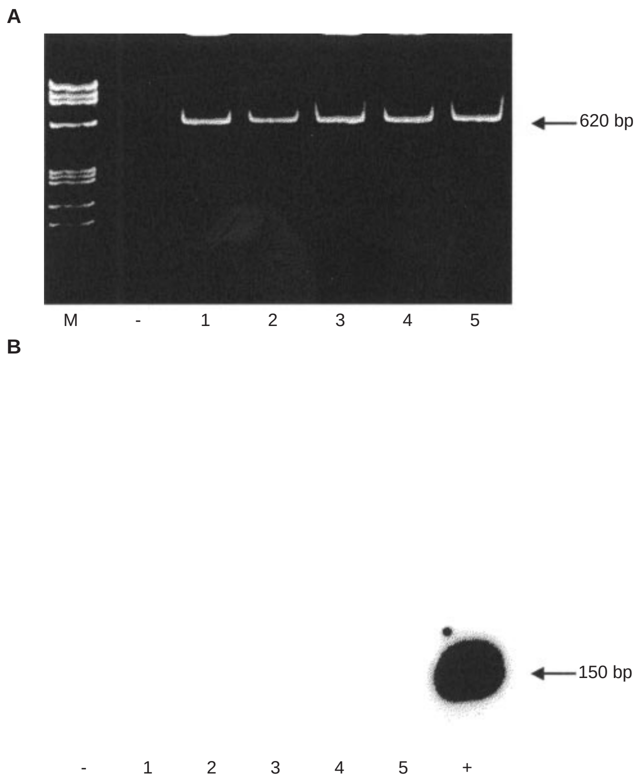

**Figure 1** PCR amplification of representative cALL samples using β-globin or JC virus primers. (**A**) Ethidium bromide-stained gel showing amplification with control β-globin primers which amplify a fragment of 620 bp. (**B**) Autoradiograph obtained following amplification with JC primers and hybridization with a virus-specific probe. M, DNA size marker, Hae III-digested φ X174; –, negative control, water; 1–5, DNA from representative leukaemic samples; +, positive control, JC virus-positive urine sample. Bp, base pairs

### RESULTS AND DISCUSSION

No positive results were obtained using the JC and BK virus primers and the cALL samples. All samples contained DNA amplifiable with the β-globin primers and positive controls gave consistent results (Figure 1).

The results of this study indicate that JC and BK viral sequences are not detectable in the leukaemic cells of cALL cases tested, and provide no support for the hypothesis that JC virus is directly and commonly involved in the aetiology of cALL. The data do not rule out the possibility that these viruses play an indirect role in disease pathogenesis, that they use a 'hit and run' mechanism or are involved in a small minority of cases.

### ACKNOWLEDGEMENTS

This work was supported by the Leukaemia Research Fund as part of LRF Specialist Programme grants to the LRF Virus Centre and LRF Centre at the Institute of Cancer Research. We should like to thank Chris Perrons for providing positive control samples and Alice Gallagher and Sue Colman for technical assistance. We are grateful to colleagues in the UK Children's Cancer Study Group for access to the clinical material used in this study.

### REFERENCES

Greaves MF (1997) Aetiology of acute leukaemia. *Lancet* **349**: 344–349

Greaves MF and Alexander FE (1993) An infectious etiology for common acute lymphoblastic leukemia in childhood. *Leukemia* **7**: 349–360

Kinlen LJ (1995) Epidemiological evidence for an infective basis in childhood leukaemia. *Br J Cancer* **71**: 1–5

Kinlen LJ, Clarke K and Hudson C (1990) Evidence from population mixing in British New Towns 1946–1985 of an infective basis for childhood leukaemia. *Lancet* **336**: 577–582

Perrons CJ, Fox JD, Lucas SB, Brink NS, Tedder RS and Miller RF (1996) Detection of polyomaviral DNA in clinical-samples from immunocompromised patients: correlation with clinical disease. *J Infect* **32**: 205–209

Saiki RK, Gelfand DH, Stoffel S, Scharf SJ, Higuchi R, Horn GT, Mullis KB and Erlich HA (1988) Primer-directed enzymatic amplification of DNA with a thermostable DNA polymerase. *Science* **239**: 487–491

Smith M (1997) Consideration on a possible viral etiology for B-precursor acute lymphoblastic leukemia of childhood. *J Immunother* **20**: 89–100

**Table 1** Primers and probes used in PCR experiments

| Virus/Gene | Oligonucleotide | Nucleotide sequence | Position* |
|---|---|---|---|
| JC virus | Forward primer | AAGTCTTTAGGGTCTTCTACCT | 4254–4275 |
| | Reverse primer | ATGGGAATCCTGGTGGAATACA | 4403–4382 |
| | Probe | CTTCATGGCAAAACAGGTCTTCATCCCACT | 4342–4371 |
| BK virus | Forward primer | AAGTCTTTAGGGTCTTCTACCT | 4391–4412 |
| | Reverse primer | CTGCAATGGTGGGTCCAAAT | 4691–4672 |
| | Probe | AGAATCTGCTGTTGCTTCTTCTTCATCATCACTGGC | 4444–4473 |
| β-globin | Forward primer | ACACAACTGTGTTCACTAGC | |
| | Reverse primer | CTGAGACTTCCACAGTGATG | |

*Nucleotide positions are given with respect to the JC virus Mad1 strain (Frisque et al, 1984) and BK virus Dunlop strain (Seif et al, 1979). β-globin primers amplify a fragment of 620 base pairs; the forward primer was described previously by Saiki et al, 1988.

*© 1999 Cancer Research Campaign*

